# Fragile X Syndrome in a Female With Homozygous Full-Mutation Alleles of the FMR1 Gene

**DOI:** 10.7759/cureus.16340

**Published:** 2021-07-12

**Authors:** Farzane Vafaeie, Masoome Alerasool, Nasrin Kaseb Mojaver, Majid Mojarrad

**Affiliations:** 1 Medical Genetics Laboratory, Genetic Foundation of Khorasan Razavi, Mashhad, IRN; 2 Department of Medical Genetics, Mashhad University of Medical Sciences, Mashhad, IRN; 3 Genetic Research Center, Faculty of Medicine, Mashhad University of Medical Sciences, Mashhad, IRN

**Keywords:** fragile x syndrome, fmr1 gene, cgg repeats, g-banding karyotype, mental retardation

## Abstract

Fragile X syndrome (FXS) has been reported as the leading cause of mental retardation (MR) that predominantly involves males compared to females. An over-expansion of CGG repeats in the 5′ untranslated region of the FMR1 gene plays the primary role in this disease. In this study, we encountered a homozygote female patient affected by FMR1 expansion mutation. Surprisingly, she had inherited her full-mutated alleles from two different ancestors. This condition is an extremely rare case of FXS. After accurate genetic counseling, family members were referred to the laboratory for genetic testing. Karyotype with two X chromosomes was the finding after the G-banding study of the proband.

Molecular analysis indicated that she was a female with full-mutated or pre-mutated alleles on both of her X chromosomes. It is a rare phenomenon that we detected in this patient. We have concluded that a combination of allele instability during oogenesis and inheritance of two alleles are the leading cause of MR in the presented case.

## Introduction

Fragile X syndrome (FXS, OMIM 300624), as the most common form of inherited neurodevelopmental disorder causing mental retardation (MR) and autism spectrum disorders, has been reported as the main cause of male MR in all races and ethnic groups. This X-linked dominant disease affects approximately 1:4000 males and 1:8000 females around the world. Meanwhile, MR and other clinical symptoms are considerably milder in affected female patients than males. The hemizygous feature of the X chromosome in males and a high density of the genes playing critical roles in central nervous system development on this chromosome could be reasonable explanations for this discrepancy [1]. An over-expansion of CGG repeats in the 5′ untranslated region (5′UTR) of the FMR1 gene, located on Xq27.3, is the primary genetic mechanism that explains the disease etiology. The expansion can be defined as four allelic forms, depending on the number of CGG repeats: normal allele with 6 to 40 repeats, intermediate allele with 41 to 60 repeats, pre-mutation with 61 to 200 repeats, full-mutated allele with more than 200 repeats [2].

The 5′ UTR CGG triplet repeats are the target site of DNA methylation in the mammalian genome. Therefore, full-mutated alleles impose a large amount of methylation in just the upstream region of FMR1 gene [3,4]. Due to the hyper-methylation condition and the following gene silencing, a dramatic reduction of FMR1 protein (FMRP) production occurs. FMRP is a critical protein for normal brain development, so FMRP loss or reduction leads to a wide range of autistic phenotypes [5,6]. Affected males suffer from moderate to severe MR and some specific behavioral patterns, playing as the main hallmarks after puberty. Intellectual disorder, puffiness around the eyes, soft skin, hypotelorism, prominent ears, long palpebral fissures, broad philtrum, facial hypotonia, macroorchidism, hyper-flexible joints, and autism-like behavior are some of the other main features of the patients [1,7].

Studies have illustrated that, in contrast to the nature of dominant X-linked disease, only some heterozygous females with one full-mutated FMR1 allele show MR. At the same time, all the male patients suffer from a range of MR [8]. Intense skewed X chromosome inactivation in female patients could be the best explanation for this finding. In this regard, several studies have proved that the X inactivation ratio in FXS-affected patients skewed from 50%:50% toward 30%:70% with preference inactivation of the mutated X chromosome. Consequently, skewed X inactivation moderates the phenotype of fragile X in females [9]. Both males and females could complain from fragile-X-associated tremor/ataxia syndrome (FXTAS [MIM 300623]) as a direct consequence of FMR1 pre-mutation state; furthermore, premature ovarian failure (EXPOF [MIM 311360]) occurred in approximately 20% of females who carry a pre-mutated allele [10,11]. Pre-mutated allele expansion to full-mutated allele is identified in *de novo* cases of FXS. In several studies, the stretch of pre-mutated allele repeats has been confirmed during transmission from mothers to their offspring. This transformation of the pre-mutations occurs through oogenesis and has rarely been reported during spermatogenesis [12]. However, females carrying full-mutated FMR1 might be suffering from mild autism-like behavior, anxiety, socio-emotional difficulties and learning problems, while homozygote females with two full-mutated alleles are supposed to be affected with fundamental aspects of the disease [9]. In this report, we present a female fragile X-affected patient with two full-mutated FMR1 genes that originated from two different ancestors.

## Case presentation

**Figure 1:**
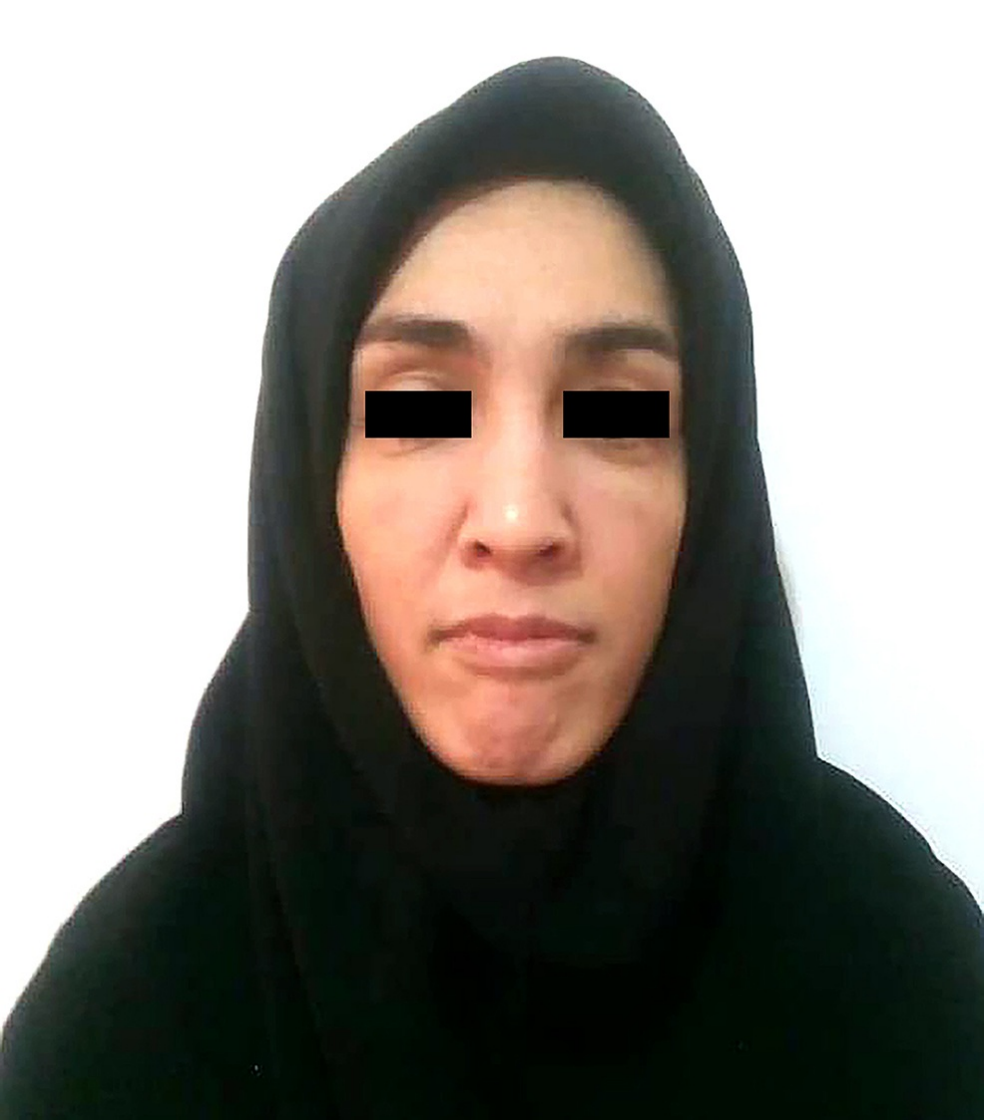
Image of the proband shows an elongated face due to a long forehead and a prominent chin

**Figure 2:**
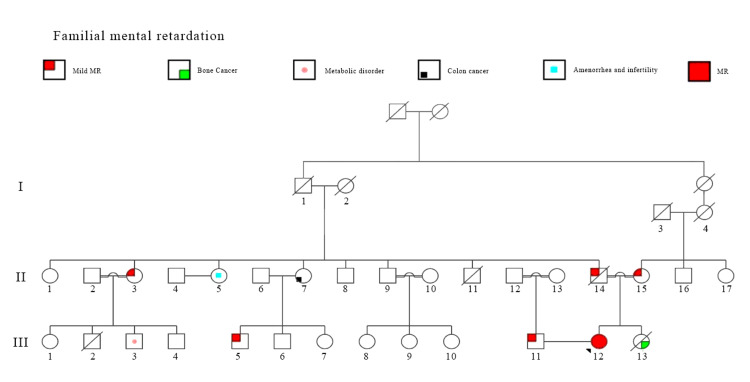
Family pedigree of the proband The arrow indicates the proband (case III12) with MR phenotype. The parents (case II14, II15), paternal aunt (case II3) and one cousin (case III5) suffered from mild MR. Three consanguinities are seen in the family. MR, mental retardation

The patient was a 37-year-old female who had an intellectual disability with fertility problems (Figure 1). As shown in Figure 2, she was the first offspring of a consanguineous couple who manifested mild MR. The proband's sister had passed away of bone cancer at the age of 32. One of her paternal aunts and one of her paternal cousins were suffering from mild MR too. After the precise genetic counseling, family members were referred to the Genetic Foundation of Khorasan Razavi for genetic testing.

According to the physical manifestation of proband's father, who had died a few years earlier, we suspected that he had been affected by FXS. Accordingly, the proband, her mother and other family members were examined for FXS using triplet-primed polymerase chain reaction (TP-PCR). As a brief, DNA extraction from peripheral blood samples was done using DNA isolation kit (Roche DNA Isolation Kit for Cells and Tissues, catalog no. 11814770001; Roche, Berlin, Germany). DNA qualification and quantification were examined using the Epoch Nanodrop UV-VIS Spectrophotometer (BioTek Instruments, VT, USA). Analysis of CGG repeats and detection of alleles with >200 repeats were done, and female zygosity was also resolved utilizing the TP-PCR method and FastFraX™ FMR1 Sizing Kit (catalog no. F2-050-V; BioFactory Pte. Ltd., Singapore), followed by fragment analysis with the Applied Biosystems Genetic Analyzer (Thermo Fisher Scientific, MA, USA). Furthermore, TP-PCR was carried out on a paraffin-embedded tissue sample of the dead sister of the proband.

**Figure 3:**
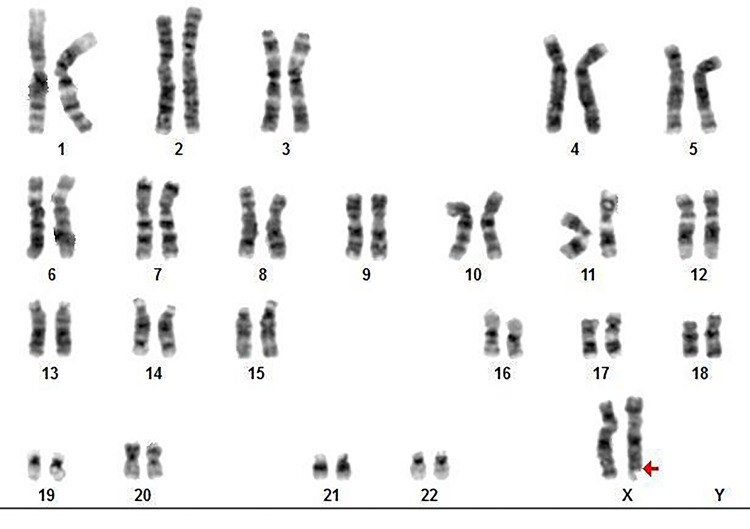
G-banding karyotype (≥500 bands) showing apparently a normal X and fragile X [fra(X)(q27.3)] chromosomes (shown by a red arrow), in case III12 in the pedigree

The patient and her husband were examined cytogenetically using the G-banding karyotype analysis method. Proband karyotype analysis using the G-banding technique indicated a female karyotype representing several spreads with one fragile X chromosome beside a normal copy of the X chromosome (Figure 3) and her husband revealed a normal karyotype. It is well known that G-banding karyotype analysis method is a complementary test and it cannot detect all fragile X chromosomes.

**Figure 4:**
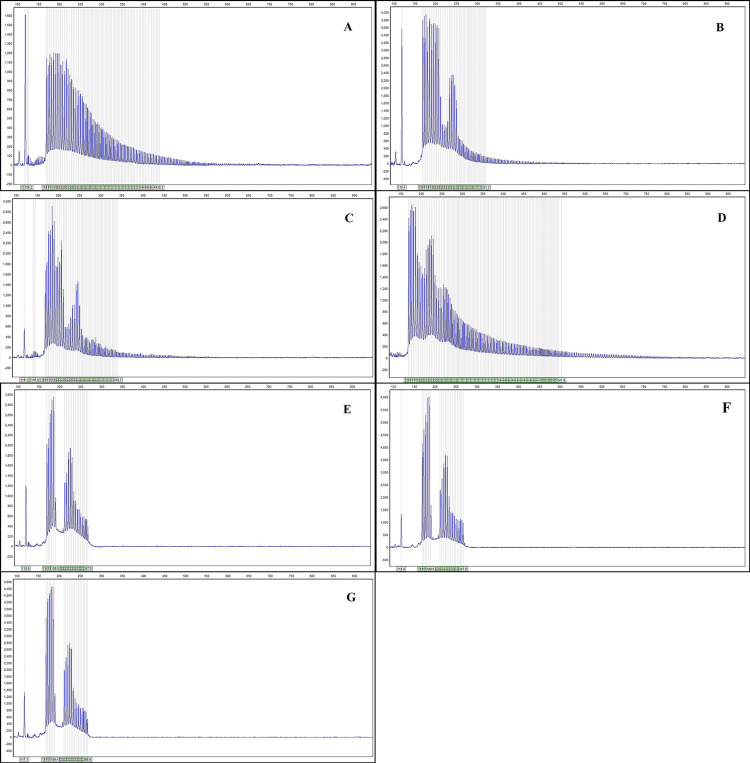
Graphs showing the number of CGG repeats in different members of the family: (A) case III12, (B) case II15, (C) case III13, (D) case II3, (E) case III8, (F) case III9, (G) case III10

Molecular analysis brightly showed two full-mutated alleles (>200 CGG) in the proband (case III12) (Figure 4A). The proband's mother (case II15) had a mild MR phenotype and was a heterozygous carrier of one pre-mutated allele (Figure 4B). Analysis of extracted DNA from the paraffin-embedded tissue (case III13) detected a heterozygote of pre-mutation expansion (Figure 4C). Although the molecular analysis of the father (case II14), who had passed away, was impossible, based on the investigation, we realized that he also had been suffering from mild MR. Proband's paternal aunt (case II3), with one normal and one full-mutated allele resulting in a mild MR, had a normal daughter and three sons (Figure 4D). One of them passed away in early childhood because of infectious disease, the other suffers from metabolic disorder (hyperkalemic periodic paralysis), and the last son is normal. To examine the expansion of the repeats in some other family members, we analysed the expansion in three of the proband's cousins (III8, III9, and III10). All of them had the normal FMR1 alleles (Figures 4E-4G). Table 1 shows the CGG repeats and mutation state in family members.

**Table 1:** Results of diagnostic testing NL: normal alleles with 6-40 repeats; PM: pre-mutation with 61-200 repeats; FM: full mutation with more than 200 repeats.

Cases	CGG repeats	Mutation state
Case III12	>200	FM
Case II15	136/25	PM carrier
Case III13	132/24	PM carrier
Case II3	245/29	FM carrier
Case III8	29/26	NL
Case III9	30/29	NL
Case III10	28/29	NL

With the analysis done in case III13, it could be concluded that the pre-mutated alleles are the unchanged pre-mutated alleles transferring from parents; in contrast, in the proband (case III12), the full-mutated alleles were the result of the repeat expansion through pre-mutated alleles' inheritance.

## Discussion

FXS can be diagnosed in both genders, while males are predominantly affected. Affected men by FXS mostly show MR and different ranges of autistic features and developmental delay, whereas females with nonspecific clinical features often have a less severe degree of the same disorders as in males. Owing to the presence of the second X chromosome with a healthy FMR1 gene in females, occasionally mild features are the only phenotypes that could be diagnosed in them even when they carry a pre-mutated or full-mutated FMR1 gene [13,14]. Recently Tabolacci et al. presented several FXS patients with fully methylated pre-mutated alleles [15]. This epigenetic finding probably could be the main reason for the mild MR observed in females with the pre-mutated FMR1 gene. In our study, we have reported a family with females, who were carriers of either pre-mutated (case II15) or full-mutated FMR1 (case II3), and manifested mild MR. However, a controversial finding that has commonly been reported in many studies has described females with a full mutation in the FMR1 gene without remarkable MR. Various features of females with a full-mutated FMR1 gene are caused by extremely skewed X-chromosome inactivation [16]. In this study, according to the cytogenetic analysis of proband's chromosomes and triplet-primed PCR result, we have found that the proband had FXS with a full-mutated FMR1 gene in both of her X chromosomes. Some other studies have determined that some pre-mutated alleles (alleles with 61-200 repeats) are unstable and could be potential precursors of the full mutation and extend through transmission from the carrier mother to the offspring during oogenesis [14,17]. The full-mutated FMR1 alleles in the proband could be the result of the highly unstable pre-mutated FMR1 allele of her mother that is expanded through oogenesis; on the other hand, the proband's father had mild MR phenotype, and based on her paternal aunt (case II13) molecular analysis result, it can be hypothesized that the father could have carried a pre-mutation or full mutation on the FMR1 gene. Therefore, the proband as the offspring of the pre-mutated mother carrier and described father would carry a significant risk of being affected as a homozygous, carrying pre-mutation or full mutation [18,19]. Compound heterozygous female patients result from parents with two mutated alleles from a different origin. In comparison, female patients with homozygous expansion in the FMR1 gene are extremely rare. This problem often occurs due to the consanguineous marriage of an affected male with his carrier cousin; therefore, consanguinity and inbreeding raises the risk of X-linked syndromes in females, as the situation increases the risk of common alleles in parents [20]. In 2020, Kim and colleagues described a three-year-old girl with typical FXS, with fully expanded FMR1 alleles (288 CGG repeats), resulting from uniparental isodisomy of the X chromosome transmitted from her mother. The latter carried a pre-mutated allele [14]. However, there are few reports on homozygous expansion in the FMR1 gene associated with FXS in females.

## Conclusions

Due to the non-typical dysmorphic features and autism or autistic behavior in females affected with FXS, the diagnosis of FXS should be considered during genetic counseling of every female with a mental disability. Our case report indicates the importance of FMR1 gene analysis in females with intellectual disability or a family history of FXS. The possibility of being a carrier of FMR1 abnormal alleles for both parents should also be considered, especially if they are consanguineous or originated from a small and isolated community.
